# Disinhibition Mediates a Form of Hippocampal Long-Term Potentiation in Area CA1

**DOI:** 10.1371/journal.pone.0007224

**Published:** 2009-09-29

**Authors:** Jake Ormond, Melanie A. Woodin

**Affiliations:** Department of Cell & Systems Biology, University of Toronto, Toronto, Ontario, Canada; The Research Center of Neurobiology - Neurophysiology of Marseille, France

## Abstract

The hippocampus plays a central role in memory formation in the mammalian brain. Its ability to encode information is thought to depend on the plasticity of synaptic connections between neurons. In the pyramidal neurons constituting the primary hippocampal output to the cortex, located in area CA1, firing of presynaptic CA3 pyramidal neurons produces monosynaptic excitatory postsynaptic potentials (EPSPs) followed rapidly by feedforward (disynaptic) inhibitory postsynaptic potentials (IPSPs). Long-term potentiation (LTP) of the monosynaptic glutamatergic inputs has become the leading model of synaptic plasticity, in part due to its dependence on NMDA receptors (NMDARs), required for spatial and temporal learning in intact animals. Using whole-cell recording in hippocampal slices from adult rats, we find that the efficacy of synaptic transmission from CA3 to CA1 can be enhanced without the induction of classic LTP at the glutamatergic inputs. Taking care not to directly stimulate inhibitory fibers, we show that the induction of GABAergic plasticity at feedforward inhibitory inputs results in the reduced shunting of excitatory currents, producing a long-term increase in the amplitude of Schaffer collateral-mediated postsynaptic potentials. Like classic LTP, disinhibition-mediated LTP requires NMDAR activation, suggesting a role in types of learning and memory attributed primarily to the former and raising the possibility of a previously unrecognized target for therapeutic intervention in disorders linked to memory deficits, as well as a potentially overlooked site of LTP expression in other areas of the brain.

## Introduction

Plasticity of synaptic connections between neurons in the hippocampus is thought to play a central role in learning and memory. Synaptic plasticity can be induced by patterned electrical stimulation at a number of synapses in the hippocampus, including the excitatory synapses of the trisynaptic and direct entorhinal-CA1 pathways, as well as at certain excitatory onto interneuron synapses, and inhibitory onto pyramidal neuron synapses [see 1 for a review]. LTP of CA3-CA1 glutamatergic transmission has become the leading model of synaptic plasticity, in part because of its dependence on NMDAR activation [2], which provides a mechanism for associating pre- and postsynaptic action potential firing, and which is also required *in vivo* for hippocampal-dependent spatial and temporal learning [3]–[5].

An analysis of the literature on feedforward inhibition in CA1 suggests that plasticity at inhibitory synapses might also be able to play a role in regulating the efficacy of CA3-CA1 transmission. When presynaptic CA3 pyramidals fire, the EPSP recorded in CA1 is followed in less than 2 ms by a disynaptic IPSP [6] originating from basket cells targeting the somatic compartment [7]. This delay between EPSP and IPSP is only half as long as the rise time of unitary EPSPs evoked by single cell firing in CA3 [8]. Furthermore, feedforward inhibition has been shown to overlap with the rising phase of the EPSP in hippocampal slices from guinea pigs [9]. Thus, feedforward inhibition should reduce EPSP amplitude recorded at the soma, as demonstrated for unitary EPSPs between pairs of CA3 neurons [10]. It follows that disinhibition, if expressed at feedforward synapses, would reduce the shunting of excitatory currents, leading to an increase in EPSP amplitude.

**Figure 1:**
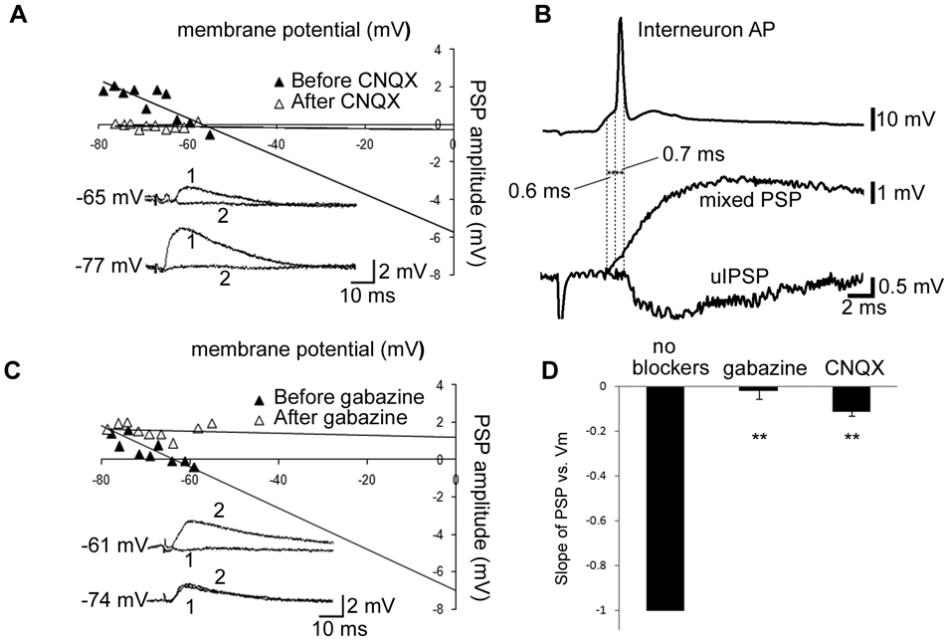
Feedforward inhibition reduces EPSP amplitude. A, PSP amplitude vs. V_m_ for one cell before and after CNQX. Inset: Sample traces before (1) and after (2) CNQX application. B, sample traces demonstrating the delay between the evoked pyramidal PSP and interneuron AP, and the delay between the interneuron AP and unitary IPSP (note that the intracellularly evoked interneuron AP, which produced the uIPSP, has been omitted for clarity). The PSP was recorded from a membrane potential of −82 mV; the uIPSP was recorded from a membrane potential of −48 mV. C, left, PSP amplitude vs. V_m_ for one cell before and after gabazine. Inset: Sample traces before (1) and after (2) gabazine application. D, The slope of the PSP vs. Vm graph before and after either gabazine (n = 4) or CNQX (n = 29), normalized to the before drug slope. ** denotes statistical significance from ctrl (no blockers; ** p<0.001). Membrane potential denoted to left of sample traces in A and C.

While a number of studies have reported the expression of activity-dependent reductions in strength at inhibitory synapses in area CA1 [11]–[14], plasticity specifically at the feedforward inhibitory synapses has never been demonstrated. The purpose of the present study was to determine if pairing-induced disinhibition at feedforward inhibitory synapses would alter the efficacy of CA3-CA1 excitatory transmission. Being careful not to directly stimulate inhibitory fibers (see Figure 1A and D, Figure S1) while making whole-cell recordings in hippocampal slices from 2 month old rats, we found that feedforward inhibition does indeed reduce EPSP amplitude, and that disinhibition at feedforward synapses, expressed as a depolarization of the reversal potential for GABA_A_R-mediated currents [14] contributes to the increase in EPSP amplitude seen during LTP expression. Furthermore, we found that disinhibition can produce LTP of Schaffer collateral-mediated transmission even under conditions in which classic LTP at glutamatergic synapses is not expressed. Taken together, our results suggest that plasticity of feedforward GABAergic synapses may play a similar role to classic LTP in memory encoding in the hippocampus.

## Materials and Methods

### Ethics statement

All rats were maintained on a 12 h light/dark cycle with food and water provided ad libitum. The Animal Studies Committee at the University of Toronto approved all experimental protocols.

### Brain slice preparation

400 µm hippocampal slices were prepared from 50–75 day old (except where noted) male Sprague Dawley rats anaesthetized with a mixture of ketamine and xylazine, and perfused through the ascending aorta with chilled modified artificial cerebrospinal fluid (ACSF). Modified ACSF was composed of 180 mM sucrose, 25 mM sodium bicarbonate, 25 mM glucose, 2.5 mM KCl, 1.25 mM sodium phosphate, 2 mM MgCl_2_, 1 mM CaCl_2_, 0.4 mM sodium ascorbate, and 3 mM sodium pyruvate, and saturated with 95% O2/5% CO2 (pH 7.4, osmolarity ∼305 mOsm). After cardiac perfusion, the brain was quickly removed, and hemispheres were separated and placed into the chilled solution for another 30 seconds. Hippocampi were then partially isolated by removing the cerebellum and all cortex except that directly overlying the hippocampus, to avoid damaging area CA1 while increasing the hippocampal surface area contacting oxygenated solution. The hippocampi were mounted vertically on an agar block and 400 µm slices cut with a Vibratome 1000 plus. Slices recovered in 35–37°C ACSF composed of 125 mM NaCl, 25 mM glucose, 25 mM sodium bicarbonate, 2.5 mM KCl, 1.25 mM sodium phosphate, 1 mM MgCl_2_, and 2 mM CaCl_2_ and saturated with 95% O2/5% CO2 (pH 7.4, osmolarity ∼305 mOsm) for 1 hour.

### Electrophysiology

Whole-cell recordings were made in oxygenated ACSF at 35–37°C from CA1 pyramidal cells, and in some experiments, presynaptically connected feedforward interneurons. Pyramidal cells were identified by the presence of an after-depolarization following action potential firing, as well as action potential accommodation during prolonged AP trains. Feedforward interneurons were recorded in the pyramidal cell layer. Generally, we targeted cells that had much larger, and more irregularly shaped, cell bodies than the pyramidal cells. Their identity was confirmed electrophysiologically; they were excited to threshold by relatively low levels of Schaffer collateral stimulation (blocked by CNQX), and inhibited CA1 pyramidal neurons. Intracellular current injection always produced APs with large after-hyperpolarization; firing was always non-accommodating.

Whole-cell recording pipettes were pulled from thin-walled borosilicate (World Precision Industries, TW-150F) with a Sutter Instruments P-87 to resistances of 5–8 MOhms. Pipettes were filled with a solution consisting of 130 mM potassium gluconate, 10 mM KCl, 10 mM HEPES, 0.2 mM EGTA, 4 mM ATP, 0.3 mM GTP, 10 mM phosphocreatine (pH 7.25, osmolarity 275–285 mOsm). IPSPs recorded with this intracellular solution reversed at −88.3±1.6 mV (LJP corrected; −74.3 mV uncorrected; n = 12). This was nearly identical to the reversal recorded with gramicidin perforated patch (−88.5±1.2 mV LJP corrected; −87 uncorrected; n = 6, data not shown), and there was no statistical difference between the two groups (p = 0.673). All membrane potential values in the text and figures are uncorrected for the liquid junction potential. Signals were amplified using an Axon Instruments Multiclamp 700b and digitized using an Axon Instruments Digidata 1322a. The bridge was balanced upon going whole-cell, and then monitored and adjusted as necessary throughout the duration of recording.

**Figure 2:**
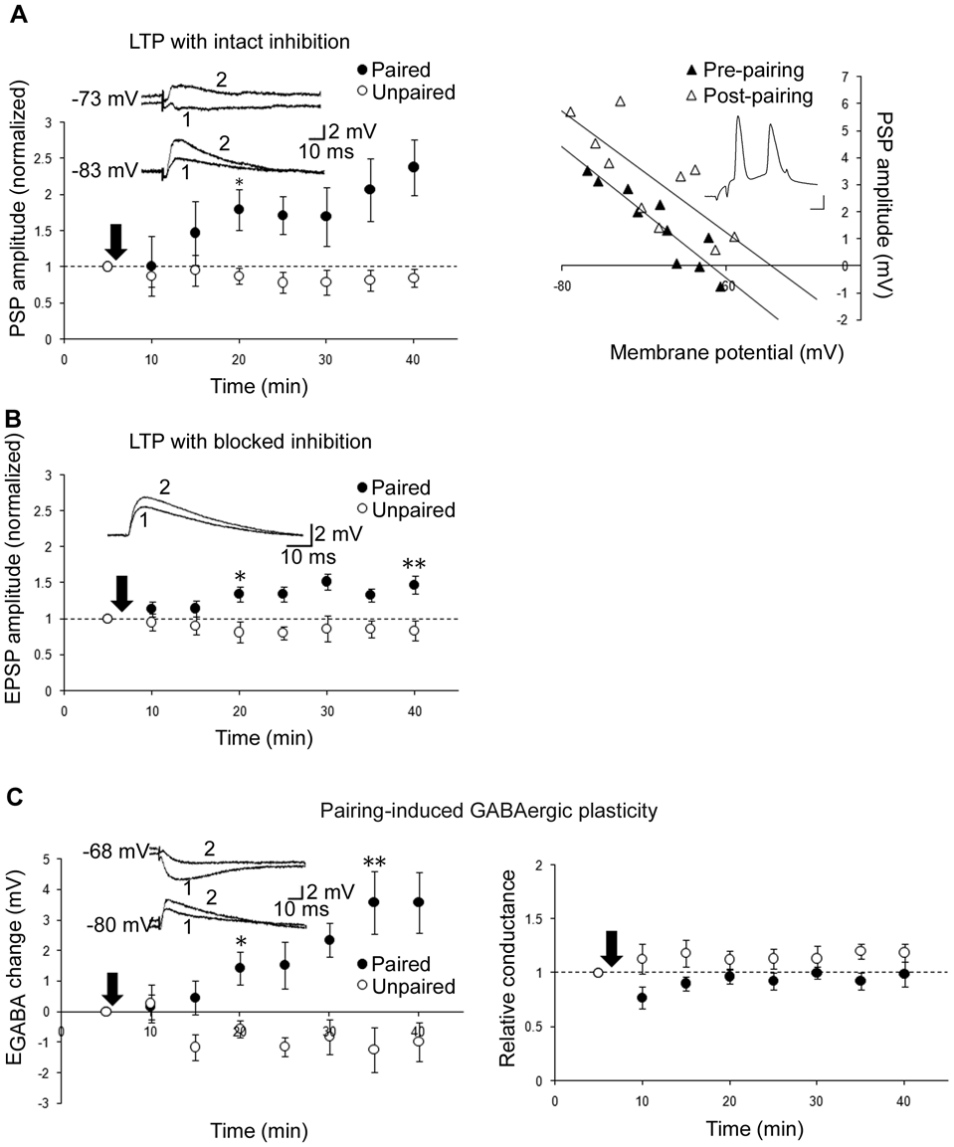
E_GABA_ depolarization underlies a large component of mixed LTP. A, Left: Mixed LTP (pairing n = 6, ctrl n = 5). Inset: Sample traces before (1) and after (2) paired activity at two holding potentials. Right: Sample PSP vs. membrane potential plots for one cell before and 35 min after pairing. Inset: example of one pairing demonstrating timing of presynaptic stimulation and action potential firing. Scale bars: 20 mV, 2 ms. B, glutamatergic LTP (pairing n = 7, ctrl n = 5; gabazine present throughout recording). Inset: Average of 10 traces before (1) and after (2) paired activity. C, GABAergic plasticity: change in E_GABA_ (left) and conductance (right; pairing n = 6, ctrl n = 5, same cells left and right; CNQX present throughout recording; NMDARs left unblocked). Inset: Sample traces before (1) and after (2) paired activity, at two holding potentials. * and ** denote statistical significance from control (p<0.05 and p<0.001, respectively) from that time point on. Arrows denote plasticity induction.

Extracellular stimulation was applied through a whole-cell recording pipette containing a silver chlorided wire and filled with ACSF. The stimulus was generated by an AMPI ISO-Flex stimulus isolator triggered by an AMPI Master 8 stimulator, and was 100 µsec in duration. The baseline recording frequency was 0.0333 Hz. Plasticity was induced by pairing extracellular stimulation with simultaneous current injection (1 nA for 10 ms) at 5 Hz (300 pairings; Figure 2A right inset shows an example pairing). For experiments examining LTP of mixed glutamatergic and GABAergic transmission, stimulation was applied to the Schaffer collaterals in CA3, so as to avoid the recruitment of monosynaptic inhibition in CA1 (Figure S1A). In experiments examining GABAergic plasticity, CNQX (10 µM; Sigma) was applied to block AMPAR-mediated transmission, and the stimulating electrode was placed in stratum radiatum within 10–20 µm of stratum pyramidale (Figure S1B). In experiments examining glutamatergic LTP, gabazine (6 µM; Tocris) was applied to block GABA_A_R-mediated transmission, the CA3 region was cut away to prevent the generation of epileptic activity, and stimulation was applied in the Schaffer collaterals in CA1 (Figure S1C).

APV (25 µM; Sigma), CNQX and gabazine were applied through bath perfusion, while AIP (5 µM; Sigma) was applied through the patch pipette diluted in internal recording solution.

To determine the EPSP/feedforward IPSP delay, two measurements were made. First, the Schaffer collaterals were stimulated and the delay between the resulting PSP onset in the pyramidal neuron (determined visually) and the AP fired by the feedforward interneuron was measured. Second, AP firing in the interneuron was evoked with intracellular current injection, and the delay between the AP and the resulting postsynaptic unitary IPSP was measured (Figure 1B). These two delays were added to give the EPSP/feedforward IPSP delay.

To verify that the recorded inhibition was feedforward and not due to direct stimulation of inhibitory fibers, CNQX was perfused into the bath at the end of all mixed recordings. CNQX reduced the mean slope of the PSP vs. membrane potential relationship for all recordings by 89% (Figure 1D; Figure S2 displays this data for each individual experimental group). Because glutamatergic EPSPs make no significant contribution to the slope at the membrane potentials examined (Figure 1C, D), the reduction in slope after CNQX can be attributed to reduced inhibition.

### Data Analysis

Data was acquired using Axon Instruments Clampex 9 software, and analyzed using Axon Instruments Clampfit and Microsoft Excel. In all recordings, intracellular current steps were applied simultaneously with extracellular stimulation in sequences of 10, from the most negative to the most positive, so that PSP amplitude versus membrane potential graphs could be constructed in Microsoft Excel for each 5 min segment of the recording. For GABAergic recordings, the shift in the IPSP amplitude versus membrane potential graphs' x-intercept gave the change in E_GABA_, while the change in slope provided the relative change in synaptic conductance. For mixed EPSP/IPSP recordings, the graphs were used to determine the average PSP amplitude from resting membrane potential (RMP; measured at the outset of each recording) for each 5 min segment. As EPSP amplitude showed no voltage dependence (Figure 1C, D), it was averaged for each 5 min segment to aid comparison with the other experimental groups.

### Statistics

**Figure 3:**
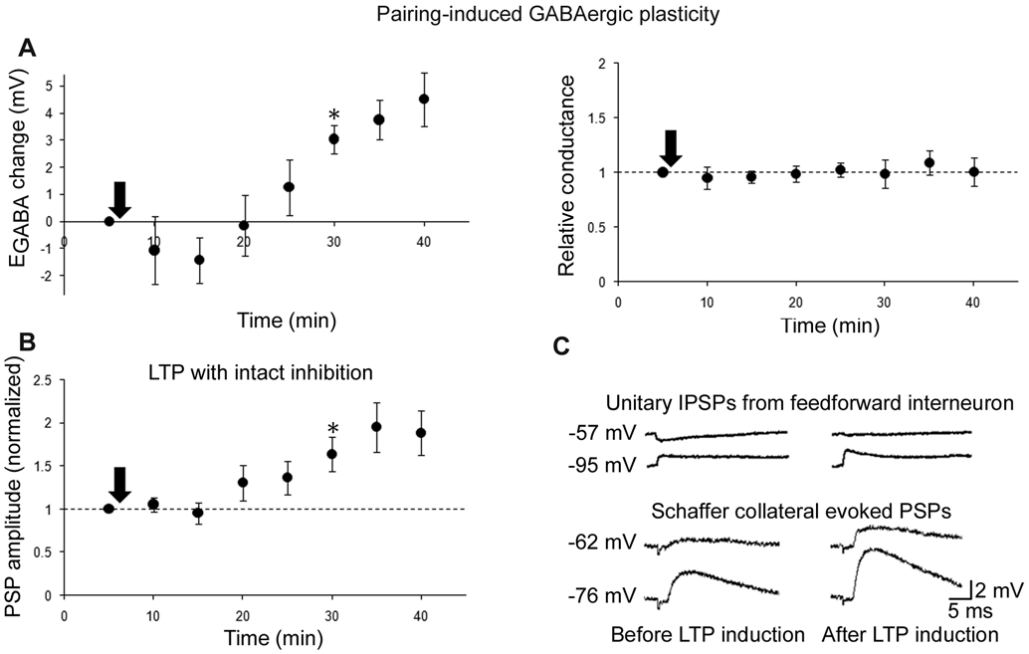
LTP induction leads to simultaneous E_GABA_ depolarization and LTP. A, GABAergic plasticity as determined by a depolarization of E_GABA_ (left) and no change in conductance (right). B, Mixed LTP with intact inhibition (n = 7). *denotes statistical significance (p<0.05) from that time point on. Arrows denote plasticity induction. C, sample traces of membrane potential from one cell at different holding potentials (denoted to left of sample traces).

**Figure 4:**
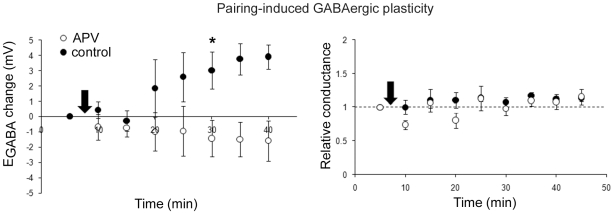
APV blocks pairing-induced GABAergic plasticity. GABAergic plasticity induced in the presence or absence of APV: change in E_GABA_ (left) and conductance (right; with APV n = 5, without APV n = 4, same cells left and right; CNQX present throughout recording). * denotes statistical significance from control (p<0.05) from that time point on. Arrows denote plasticity induction.

**Figure 5:**
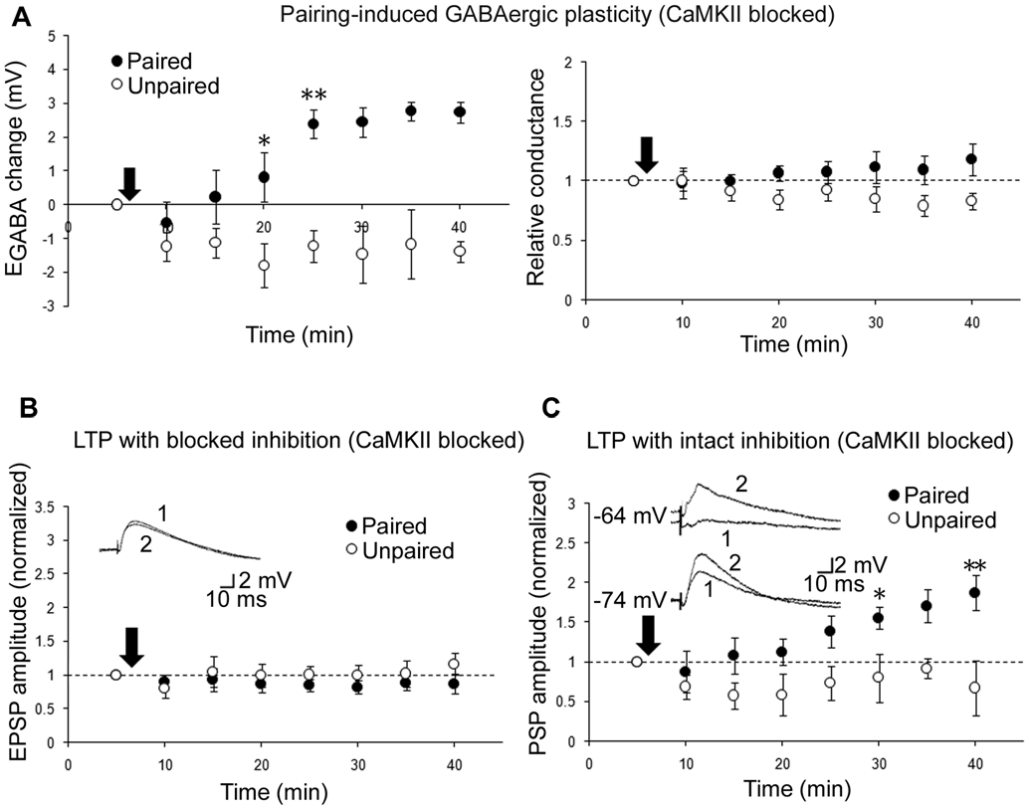
Depolarization of E_GABA_ is sufficient for LTP expression. A, GABAergic plasticity induced during CaMKII inhibition (change in E_GABA_ at left, conductance at right; pairing n = 7, ctrl n = 5, same cells left and right; CNQX present throughout recording, NMDARs left unblocked). Inset: Sample traces before (1) and after (2) paired activity. B, Glutamatergic LTP induced during CaMKII inhibition (pairing n = 5, ctrl n = 4; gabazine present throughout recording). Inset: Average of 10 traces before (1) and after (2) paired activity. C, Mixed LTP induced during CaMKII inhibition (pairing n = 6, ctrl n = 5). Insets: Sample traces before (1) and after (2) paired activity at two holding potentials (denoted to left of traces). CaMKII inhibitor AIP present in all recordings. * and ** denote statistical significance from control (p<0.05 and p<0.001, respectively) from that time point on. Arrows denote plasticity induction.

**Figure 6:**
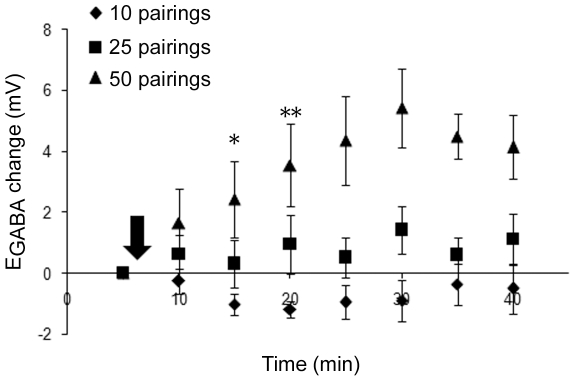
Depolarization of E_GABA_ can be induced by as few as 50 pairings. GABAergic plasticity induced by 10 pairings (diamond, n = 7), 25 pairings (square, n = 6), or 50 pairings (triangle, n = 5). CNQX present throughout recordings (NMDARs left unblocked). * denotes statistical significance from Figure 2C control (* <0.05, ** <0.001; for statistical significance between groups see text) from that time point on.

Results are expressed as mean±s.e.m. All statistical tests were performed in Sigmastat. Significance was determined using either a paired Student's t-test (Figure 1D, 3, S2) with significance level of P<0.05 or two-way repeated measures ANOVA (significance level of P<0.05) with post-hoc Tukey test (Figures 2, 4, 5, and 6; P values reflect the results of the post-hoc Tukey test). For all multiple comparisons in which statistical significance is reported, ANOVA values were significant to 0.05 or lower. EPSP 90% amplitude and rise time correlation was determined with linear regression.

## Results

To investigate the effect of feedforward inhibition on the amplitude of Schaffer collateral-mediated EPSPs, we stimulated the Schaffer collaterals close to their site of origin in CA3 (Figure S1A), and recorded mixed glutamatergic and GABAergic PSPs from pyramidal neurons in CA1. These PSPs were largely abolished by perfusion of the AMPA receptor (AMPAR) antagonist CNQX (10 µM; Figure 1A, D, Figure S2), indicating that inhibition was primarily elicited disynaptically. To determine whether the onset of feedforward inhibition was indeed rapid enough to reduce EPSP amplitude, we directly measured the EPSP-feedforward IPSP delay, previously found to be 1.9 ms in hippocampal slices from 1 month old Wistar rats recorded at 33°C [6]. To this end, we made paired recordings between synaptically connected feedforward interneurons (identified electrophysiologically; see Methods) and pyramidal cells. To calculate the EPSP-IPSP delay, the delay between the onset of the Schaffer collateral-evoked pyramidal cell EPSP and the interneuron AP (0.39±.33 ms, n = 10) was added to the delay between the intracellularly-evoked interneuron AP and the resulting pyramidal unitary IPSP [0.90±0.07 ms; same cells (n = 10); Figure 1B]. This gave an EPSP-IPSP onset delay of 1.3±.3 ms (n = 10), much briefer than the mean rise time to 90% amplitude of pharmacologically isolated Schaffer collateral-evoked EPSPs [6.1±.3 ms; no correlation was observed between 90% amplitude (range = 0.5–4.5 mV) and rise time (r^2^ = 0.00595, p = 0.623, n = 43); data not shown]. This indicated that feedforward inhibition arrives in time to reduce EPSP amplitude. Moreover we found that membrane depolarization, which increases the driving force for Cl^−^ in the inward direction, has a strongly depressive effect on EPSP amplitude (Figure 1A, C; the mean reversal potential of mixed PSPs was −62.03±1.37 mV, n = 22). This depression of EPSP amplitude was completely blocked by the GABA_A_ receptor (GABA_A_R) antagonist GABAzine (6 µM), indicating that disynaptic inhibition elicited by Schaffer collateral-mediated glutamatergic transmission decreases the amplitude of EPSPs recorded at the soma.

We next examined how feedforward inhibition affects the magnitude of pairing-induced LTP. Pairing Schaffer collateral stimulation with postsynaptic spiking (300 pairings at 5 HZ; Experimental Procedures) produced a robust increase in mixed PSP amplitude (Figure 2A). However, when feedforward inhibition was blocked with gabazine, LTP was reduced (Figure 2B). The mere presence of feedforward inhibition would not be expected to enhance LTP, and has in fact been reported to instead diminish its magnitude [15]. We therefore suspected that a reduction in inhibitory strength induced by pairing was responsible for the enhanced LTP. To determine if this might be the case, we isolated inhibition pharmacologically with CNQX (NMDARs left unblocked), and paired extracellular stimulation close to the cell body layer (the site of feedforward synapses [7]) with postsynaptic spiking. This induced a depolarization of E_GABA_ (Figure 2C), as previously reported in hippocampal cultures and slices from juvenile rat [14]. Importantly, this also confirmed the previously reported observation that whole-cell recording does not interfere with the expression of pairing-induced GABAergic plasticity [14], which results from intracellular Cl^−^ accumulation due to a Ca^2+^ dependent down-regulation of the K^+^/Cl^−^ cotransporter KCC2. No long-term changes in relative synaptic conductance were observed (Figure 2C, p = 0.173). These results suggest that pairing-induced weakening of the GABAergic driving force increases the magnitude of LTP.

We next sought a direct confirmation that E_GABA_ depolarizes during LTP expression. To this end, we induced and monitored LTP with Schaffer collateral stimulation, while simultaneously monitoring E_GABA_ by evoking APs in a synaptically connected feedforward interneuron. Indeed, both E_GABA_ depolarization (Figure 3A, C) and LTP (Figure 3B, C) were expressed after pairing. Thus, pairing Schaffer collateral stimulation with pyramidal cell spiking induces a weakening of the driving force for GABAergic currents at feedforward synapses that contributes to LTP expression.

As a final confirmation of our results, we asked whether E_GABA_ depolarization alone would be sufficient to produce LTP. Our strategy was to block glutamatergic LTP pharmacologically with a drug that would leave pairing-induced inhibitory plasticity unaffected. Previous results had ruled out the involvement of NMDARs in pairing-induced inhibitory plasticity in slices from juvenile rats [14], making NMDAR blockers an attractive choice. However, given the possibility of developmental changes in the underlying mechanisms, as well as the fact that our stimulating electrode was positioned just outside of the cell body layer and therefore would have been simultaneously activating glutamatergic synapses, we felt it prudent to test the effect of NMDAR blockade on pairing-induced E_GABA_ depolarization. Surprisingly, the NMDAR blocker APV (25 µM) completely blocked both pairing-induced GABAergic plasticity (Figure 4) and LTP under conditions of intact inhibition (data not shown). We next tested inhibition of calcium/calmodulin-dependent protein kinase II (CaMKII), a Ca^2+^-dependent kinase required for classic LTP induction [16]. Applying the CaMKII inhibitor autocamtide 2-related inhibitory peptide (AIP; 5 µM; [17]) intracellularly through the patch pipette had no effect on inhibitory plasticity (Figure 5A; change in conductance p = 0.078), but abolished glutamatergic LTP when inhibition was blocked with gabazine (p = 0.537, Figure 5B). When both inhibition and excitation were left intact, LTP induced by pairing was only slightly reduced by AIP (Figure 5C), as expected from the loss of the glutamatergic LTP component (Figure 2B). Thus, we conclude that disinhibition is sufficient to produce LTP of Schaffer collateral-mediated excitatory transmission.

Given the view that the hippocampal synaptic plasticity responsible for memory formation is thought to require only short periods of action potential firing [18], we wanted to know whether E_GABA_ depolarization could be induced by a briefer induction protocol. Leaving the frequency of stimulation unchanged, we examined the ability of 10, 25, or 50 pre- and postsynaptic pairings to induce plasticity (Figure 6). Neither 10 (p = 0.74) nor 25 (p = 0.139) pairings produced significant plasticity (no significant difference from control in Figure 2C), but 50 pairings produced a depolarization of E_GABA_ to a level indistinguishable from that induced by 300 pairings [p<0.001 compared to control (Figure 2C) and 10 pairings, p = 0.005 compared to 25 pairings].

## Discussion

To our knowledge, this is the first report that long-term weakening of feedforward connections can augment the efficacy of Schaffer collateral-mediated glutamatergic transmission (see Figure S3 for model). We took great care to make sure that the inhibition being elicited was done in a way that was physiological so as to avoid overestimating the impact of inhibition on EPSP amplitude. By activating inhibitory inputs disynaptically rather than directly, we have maintained the physiological ratio of, and delay between, excitation and inhibition, and avoided the recruitment of interneuron types not involved in feedforward inhibition. Furthermore, under our whole-cell recording conditions, the driving force for GABA_A_R-mediated currents closely matched the driving force we measured with gramicidin perforated patch recording (see Materials and Methods), which leaves the intracellular [Cl^−^] unperturbed. Lastly, we used slices from adult rats in all experiments. We believe the use of slices from adults is crucial for studying the cellular mechanisms underlying learning and memory, as ongoing neural development complicates the interpretation of data from younger animals. Highlighting this fact, we previously found that E_GABA_, measured with gramicidin perforated-patch, hyperpolarizes a further 15 mV between 3 and 7 weeks of age [19] indicating that the developmental changes involved in strengthening inhibition extend into adolescence in the rat. As such, we think our study provides strong evidence of a role for disinhibition in LTP beyond the end of development, when synaptic plasticity is thought to primarily subserve the long-term storage of information.

A role for somatic disinhibition in E–S potentiation, the increase in postsynaptic excitability which normally accompanies classic LTP, has previously been reported [12]–[13]. Our data are consistent with a role for disinhibition in this phenomenon, but suggest that disinhibition-mediated E–S potentiation may result not from a change in the excitability of the postsynaptic neuron, but rather from increased somatic depolarization as a result of disinhibition at feedforward connections. This interpretation is entirely consistent with extracellular recording of E–S potentiation which shows a disproportionate enhancement of the population spike relative to the dendritic field potential [20]–[22], but will require a protocol capable of inducing disinhibition-mediated LTP without the need for pairing, which limits the disinhibition to a single neuron. It should be noted, however, that disinhibition is but one of a number of mechanisms underlying E–S potentiation, including relative changes in the ratio of excitation to inhibition due to glutamatergic LTP [23]–[24], as well as changes in intrinsic excitability expressed under conditions of pharmacological blockade of inhibition [25]–[29].

Interestingly, while the original demonstration of pairing-induced E_GABA_ depolarization in cell culture and slices from young rats (P11–19) found a requirement for Ca^2+^ influx through L-type Ca^2+^ channels rather than NMDARs [14], we instead found here that in slices from adult rats, NMDAR activation is required. This apparent developmental change in the source of Ca^2+^ required for E_GABA_ depolarization is supported by a recent report demonstrating the NMDAR-dependence of E_GABA_ depolarization induced in mature (DIV 19–22), but not young, cultures with bath perfusion of glutamate [30]. The same report also demonstrated that low-frequency stimulation of the Schaffer collaterals in 0% Mg^2+^ could induce NMDAR-dependent E_GABA_ depolarization in CA1 pyramidals of organotypic slices (prepared from P5–8, DIV 10–16). While it might seem unlikely that extracellular stimulation applied close to the cell body layer, as in our GABAergic plasticity experiments, would lead to NMDAR activation, synaptic NMDARs are in fact present all the way up the apical dendrite to the stratum pyramidale border [31]–[32]; due to the postsynaptic spiking induced during pairing, AMPAR-mediated depolarization would not have been required for the removal of their Mg^2+^ block. Candidate molecules and pathways involved in transducing NMDAR activation to KCC2 downregulation include PKC, required for the E_GABA_ depolarization induced by 5 minutes of postsynaptic spiking alone [33], and the BDNF-TrkB pathway, implicated by studies of the dramatic decrease in KCC2 expression following the kindling of seizures *in vivo* [34]–[35]. Whether the developmental changes in the activity-dependent regulation of KCC2 involve only the source of Ca^2+^, or extend to the signalling pathways mediating this regulation is a question that will also need to be explored in future studies.

The lack of robust glutamatergic LTP in our experiments was beneficial in that it allowed us to more easily identify the contribution that disinhibition makes to LTP in the mixed EPSP/IPSP condition. Nevertheless, some might find this curious given the number of publications showing that paired pre- and postsynaptic activity, when separated with a positive (pre before post) spike-timing delay, produces robust glutamatergic LTP in CA1 pyramidal cells (eg. [36]). However, a number of publications have instead shown that such pairing induces either moderate LTP (eg. [37]), weak LTP (eg. [38]), or even long-term depression (LTD; [39]). There are a number of factors to consider when evaluating the effectiveness of a given induction protocol to induce glutamatergic LTP in addition to the pre-/postsynaptic delay: these include the number of pre- and postsynaptic spikes per pairing, the frequency of pairing, and the number of pairings. For example, while it is well known that LTD can be induced by switching the order of pre- and postsynaptic pairing so that the postsynaptic firing occurs first [37], [40], it can also be induced with pre- before post pairing if the number of pairings is greatly increased [39]. This can be partly understood due to the fact that that both LTP and LTD require calcium influx through NMDARs (reviewed by [41]). Recent work has shed more light on this issue by showing that the processes underlying LTP and LTD can occur simultaneously, and that the resulting plasticity is due to the combinatory effects of these processes [42]. Given that LTP is normally induced by 50–100 pre- before post- pairings, and LTD requires approximately 900 such pairings, it is not surprising that our induction protocol consisting of 300 pairings, occupying the middle ground, induced only a weak glutamatergic LTP (though [38] reported a similar level of LTP induced with fewer pairings).

Our demonstration here that depolarization of E_GABA_ can be induced by both relatively low (50) and relatively high (300) numbers of pairings suggests that GABAergic plasticity may in some cases be co-expressed with glutamatergic LTP, and in other cases expressed in its absence. Pairing-induced LTP is hypothesized to modulate various aspects of place cell firing in the hippocampus, and possible examples of both low and high numbers of plasticity-inducing pairings can be found in the literature. For example, the experience-dependent expansion of place fields is induced by just a few passes through a given field [18]; this level of activity would be expected to induce both glutamatergic and GABAergic plasticity. Requiring more activity is the reactivation during sleep of neurons which co-fired during waking exploration [43]; this study reported that reactivation was most robust in those neurons which fired the most number of action potentials, across an examined range of 40–280, during exploration. As discussed above, increasing the number of pairings across this range would actually decrease the magnitude of glutamatergic LTP, suggesting that this phenomenon might depend solely on GABAergic plasticity, and might in fact be disrupted by the glutamatergic LTP induced with fewer pairings.

The glutamatergic LTP hypothesis of memory has been strengthened by demonstrations of LTP-like changes accompanying memory formation *in vivo* [44], [45]. Does any such evidence exist for pairing-induced GABAergic plasticity? In fact, E_GABA_ depolarization has been demonstrated in CA1 pyramidal neurons of the dorsal hippocampus in slices cut from animals that had completed spatial memory acquisition in a water maze task [46]. In that study, no parallel changes in excitatory transmission were observed, suggesting that GABAergic plasticity alone might underlie some forms of memory. This possibility is supported by our demonstration here that disinhibition at feedforward connections is sufficient for LTP expression. Furthermore, the NMDAR-dependence of pairing-induced E_GABA_ depolarization suggests that it may be involved in forms of memory attributed until now solely to classic glutamatergic LTP [3]–[5].

## Supporting Information

Figure S1 The recording configurations. (A) The configuration for recording (R) mixed excitatory and feedforward inhibitory synaptic transmission. The stimulating electrode (S), consisting of a patch pipet with silver-chlorided wire inserted, was placed close to the site of origin of the Schaffer collaterals in CA3 so as to minimize the activation of monosynaptic inhibition in CA1. (B) The configuration for recording (R) pharmacologically isolated inhibitory transmission. The stimulating electrode (S) was placed in stratum radiatum immediately adjacent (within 10–20 µm) to the cell body layer in order to stimulate somatic inhibitory synapses (which include feedforward synapses) without physically damaging pyramidal cell bodies. (C) The configuration for recording (R) pharmacologically isolated excitatory transmission. The CA3 region was cut away from the slice to avoid the generation of epileptic activity. Stimulation (S) was applied to the Schaffer collaterals in area CA1.
(1.17 MB TIF)

Figure S2 The majority of recorded inhibition in mixed EPSP/IPSP recordings was feedforward. Relative conductance after CNQX for each experimental group. Relative conductance was taken as the slope of the PSP vs. Vm graph after CNQX application divided by the slope before application (see Fig. 1A for example). Experimental groups: 1, mixed LTP (Figure 2A); 2, mixed control (Figure 2A); 3, mixed LTP (paired recordings; Figure 3B); 4, mixed LTP with AIP (Figure 5C); 5, mixed control with AIP (Fig. 5C).
(0.20 MB TIF)

Figure S3 Disinhibition can increase the efficacy of CA3 -CA1 synaptic transmission in the absence of classic LTP expression. A) The dominant theory of classic LTP is that it is expressed mainly as an increase in AMPAR insertion at the postsynaptic side of the Schaffer collateral synapses onto CA1 pyramidal neurons. Much of the excitatory current generated by Schaffer collateral transmission is shunted by temporally overlapping feedforward transmission, such that the depolarization measured at the soma is smaller than that generated at the site of excitatory transmission in the dendrites. (B) Disinhibition at the feedforward connections can also increase the efficacy with which presynaptic CA3 pyramidals excite their postsynaptic CA1 pyramidal targets. Increased intracellular [Cl-] reduces the driving force for GABAergic currents, thereby reducing the shunt of excitatory current.
(1.07 MB TIF)
