# Supplementary figures and images for: Disinhibition Mediates a Form of Hippocampal Long-Term Potentiation in Area CA1
Source: PLoS One. 2009 Sep 29;4(9):e7224. doi: 10.1371/journal.pone.0007224 (PMC2746290; doi:10.1371/journal.pone.0007224)

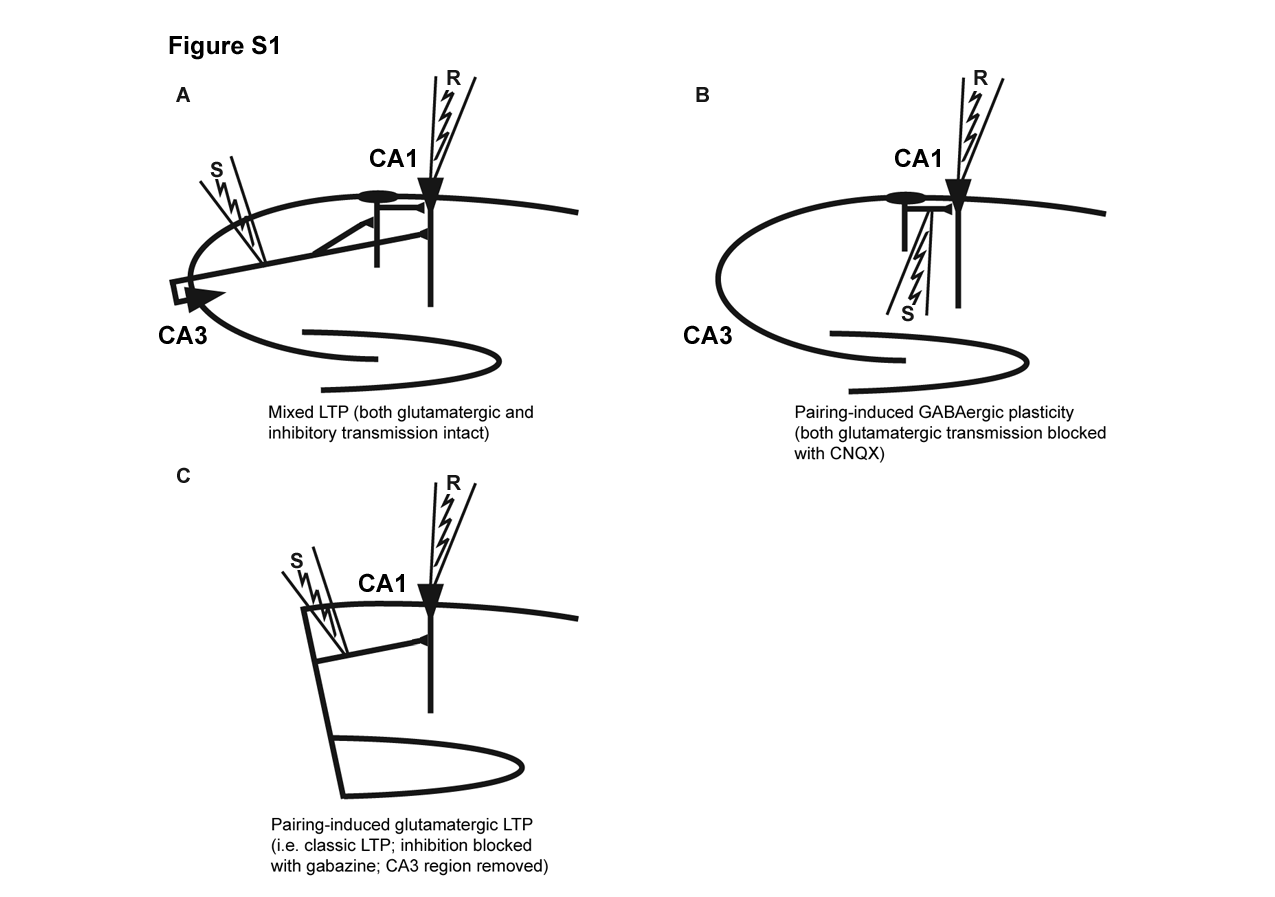

Supplement: Figure S1 — The recording configurations. (A) The configuration for recording (R) mixed excitatory and feedforward inhibitory synaptic transmission. The stimulating electrode (S), consisting of a patch pipet with silver-chlorided wire inserted, was placed close to the site of origin of the Schaffer collaterals in CA3 so as to minimize the activation of monosynaptic inhibition in CA1. (B) The configuration for recording (R) pharmacologically isolated inhibitory transmission. The stimulating electrode (S) was placed in stratum radiatum immediately adjacent (within 10–20 µm) to the cell body layer in order to stimulate somatic inhibitory synapses (which include feedforward synapses) without physically damaging pyramidal cell bodies. (C) The configuration for recording (R) pharmacologically isolated excitatory transmission. The CA3 region was cut away from the slice to avoid the generation of epileptic activity. Stimulation (S) was applied to the Schaffer collaterals in area CA1. (1.17 MB TIF) [file pone.0007224.s001.tif]

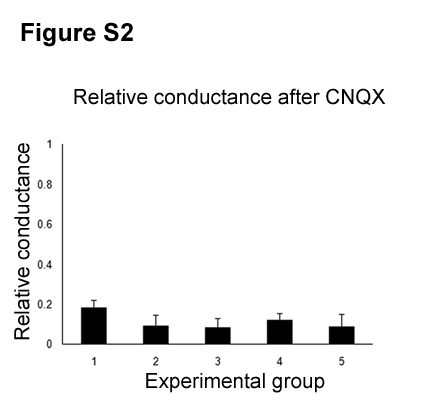

Supplement: Figure S2 — The majority of recorded inhibition in mixed EPSP/IPSP recordings was feedforward. Relative conductance after CNQX for each experimental group. Relative conductance was taken as the slope of the PSP vs. Vm graph after CNQX application divided by the slope before application (see Fig. 1A for example). Experimental groups: 1, mixed LTP (Figure 2A); 2, mixed control (Figure 2A); 3, mixed LTP (paired recordings; Figure 3B); 4, mixed LTP with AIP (Figure 5C); 5, mixed control with AIP (Fig. 5C). (0.20 MB TIF) [file pone.0007224.s002.tif]

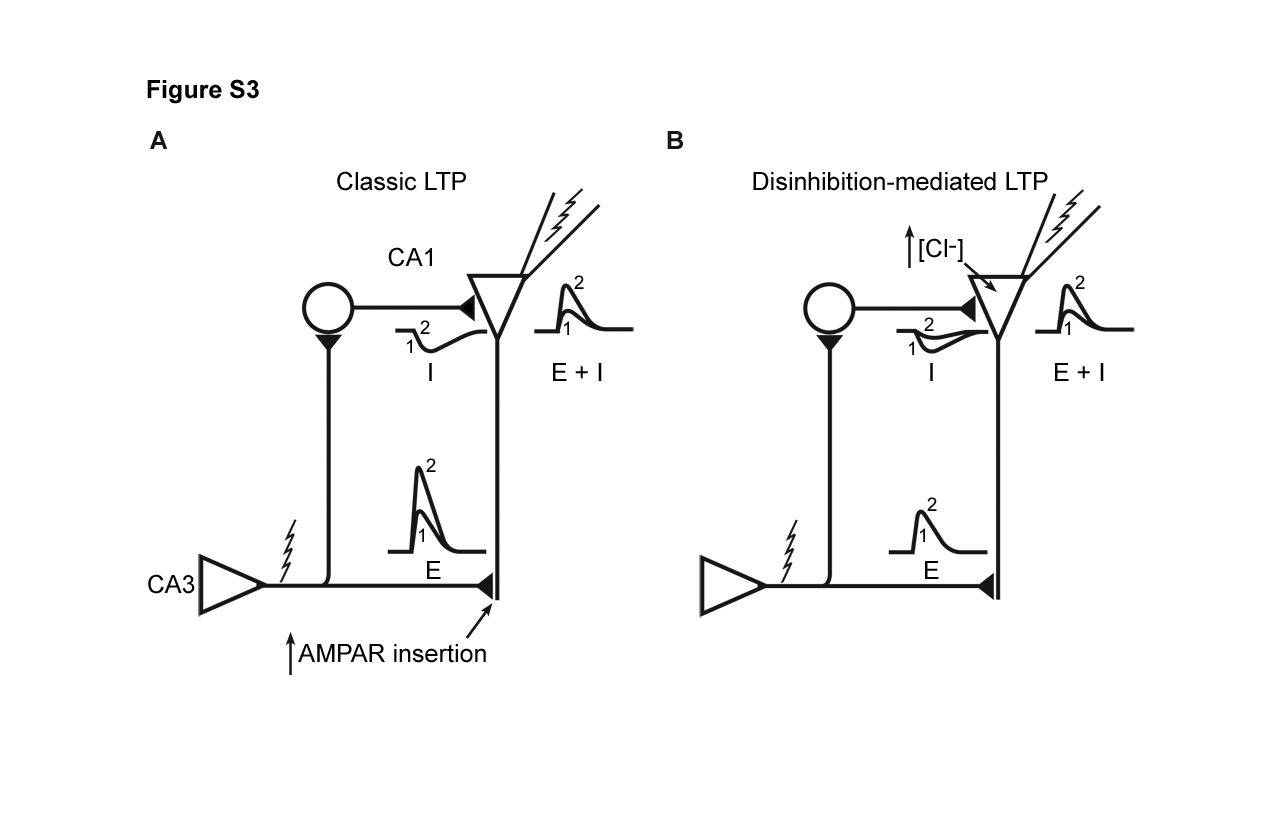

Supplement: Figure S3 — Disinhibition can increase the efficacy of CA3 -CA1 synaptic transmission in the absence of classic LTP expression. A) The dominant theory of classic LTP is that it is expressed mainly as an increase in AMPAR insertion at the postsynaptic side of the Schaffer collateral synapses onto CA1 pyramidal neurons. Much of the excitatory current generated by Schaffer collateral transmission is shunted by temporally overlapping feedforward transmission, such that the depolarization measured at the soma is smaller than that generated at the site of excitatory transmission in the dendrites. (B) Disinhibition at the feedforward connections can also increase the efficacy with which presynaptic CA3 pyramidals excite their postsynaptic CA1 pyramidal targets. Increased intracellular [Cl-] reduces the driving force for GABAergic currents, thereby reducing the shunt of excitatory current. (1.07 MB TIF) [file pone.0007224.s003.tif]
